# First time intravesically administered trifunctional antibody catumaxomab in patients with recurrent non-muscle invasive bladder cancer indicates high tolerability and local immunological activity

**DOI:** 10.1007/s00262-021-02930-7

**Published:** 2021-04-10

**Authors:** Peter Ruf, Hartwig W. Bauer, Alexandra Schoberth, Claudia Kellermann, Horst Lindhofer

**Affiliations:** 1 Trion Research GmbH, Am Klopferspitz 19, 82152 Martinsried, Germany; 2 Urologie Maximilianstrasse, Maximilianstrasse 31, 80539 München, Germany; 3 Freeline Therapeutics GmbH, Semmelweisstrasse 3, 82152 Martinsried, Germany

**Keywords:** Adjuvant intravesical treatment, Epithelial cell adhesion molecule (EpCAM), Recurrence-free intervals, Intravesical administration of Catumaxomab antibody, Recurrent non-muscle-invasive bladder cancer, Trifunctional antibody

## Abstract

Transurethral resection of the tumor (TUR-B) followed by adjuvant intravesical treatment with cytostatic drugs or Bacillus Calmette–Guérin (BCG) as standard therapy of non-muscle-invasive bladder cancer (NMIBC) is associated with a high recurrence rate of about 60–70%, considerable side effects and requires close monitoring. Alternative treatment options are warranted. Two patients with epithelial cell adhesion molecule (EpCAM)-positive recurrent non-muscle invasive bladder cancer were treated the first time by an intravesical administration of the trifunctional bispecific EpCAM targeting antibody catumaxomab (total dosage of 470 and 1120 µg, respectively). The binding and killing activity of catumaxomab in urine milieu was evaluated in vitro. In contrast to its previous systemic application catumaxomab was well tolerated without any obvious signs of toxicity. Relevant cytokine plasma levels were not detected and no significant systemic drug release was observed. The induction of a human anti-mouse-antibody (HAMA) reaction was either absent or untypically weak contrary to the high immunogenicity of intraperitoneal applied catumaxomab. Tumor cells that were detectable in urine patient samples disappeared after catumaxomab therapy. Endoscopically confirmed recurrence-free intervals were 32 and 25 months. Our data suggest that intravesical administration of catumaxomab in NMIBC is feasible, safe and efficacious, thus arguing for further clinical development of catumaxomab in this indication.

## Introduction

Bladder cancer is the 9th leading cancer entity worldwide with 430,000 new cases and about 165,000 deaths occurring yearly [1]. More than 90% of patients with bladder cancer have urothelial or transitional cell carcinoma (TCC) and the majority of cases (approximately 75%) are non-muscle-invasive (stages CIS, Ta [papillary tumor] or T1) [2]. The standard therapy of non-muscle-invasive bladder cancer (NMIBC) comprises transurethral resection of the tumor (TUR-B) followed by adjuvant intravesical treatment with cytostatic drugs or Bacillus Calmette-Guérin (BCG). However, the overall recurrence rate is high and about 60–70% of patients relapse, which requires close monitoring and continuous treatment [3]. Besides, the most effective BCG therapy causes considerable side effects in 8—20% of patients and the treatment has to be suspended due to intolerance [4, 5]. Thus, alternative treatment modalities for NMIBC are warranted.

The epithelial cell adhesion molecule EpCAM is expressed in many cancer tissues including TCC [6, 7]. Successful EpCAM-targeted immunotherapy has already been demonstrated using the antibody catumaxomab, which obtained EMA approval in 2009 for the intraperitoneal treatment of malignant ascites [8], that was—however—withdrawn in 2017 for commercial reasons. Catumaxomab is an intact trifunctional bispecific antibody that targets EpCAM and the T-cell antigen CD3. Additionally, the antibody binds and activates Fcγ R-positive accessory immune cells via its chimeric rat/mouse Fc portion [9, 10]. Consequently, EpCAM-positive cancer cells are highly efficiently killed by catumaxomab, which induces a concerted attack of T cells and accessory immune cells like monocytes, dendritic cells and NK-cells and thereby even elicits a vaccination effect [11, 12].

Although EpCAM is expressed in healthy urothelium [13], its accessibility is shielded by the glycosaminoglycan layer (GAG) that covers the luminal surface of the urothelium [14, 15]. Thus, specific uptake of the radiolabeled anti-EpCAM mouse antibody AUA1 was only shown in transitional cell carcinoma but not in healthy urothelial tissue in a biodistribution study when the antibody was administered intravesically [15]. Importantly, in this study only unremarkable antibody levels were detected in blood. Therefore, we reasoned that the intravesical route of application should prevent significant systemic exposure to catumaxomab resulting in an improved tolerability. Based on its local tumor specificity, intravesically administered catumaxomab might represent a new immunotherapeutic approach for the treatment of recurrent NMIBC. For verification of this hypothesis, we first evaluated the binding and killing activity of catumaxomab to immune and bladder cancer cells in urine milieu in vitro. Finally, two patients with recurrent NMIBC were individually treated with intravesically administered catumaxomab. Tolerability, systemic antibody exposure, human anti-mouse-antibody (HAMA) induction, cytokine plasma levels and tumor cell count in urine samples were monitored and frequent endoscopic re-evaluations were performed.

## Materials and methods

### Patients and treatment

Patients were individually treated with catumaxomab (LINDIS Biotech, Martinsried, Germany) on a named-patient basis at the urology practice *Urologie Maximilianstraße* (Munich, Germany) after obtaining written informed consent. Catumaxomab was administered to the empty bladder by a transurethral catheter in 40 ml PBS solution (pH 7.4) and held for at least two hours before voiding to allow binding of the antibody. One treatment cycle comprised six to seven weekly instillations with increasing antibody dosages between 20 and 100 µg. The dosage was chosen according to the experience gained from intraperitoneal usage of catumaxomab where the maximum tolerated dose (MTD) was defined at 10-20-50 and 200 µg [30]. This dosage approach was further justified considering the lower permeability of the bladder wall in comparison to the peritoneum and the short exposure time of only 2 h after which the drug is voided again.

### Evaluation of in vitro antibody binding and killing activity in urine milieu

Antibody binding was evaluated by flow cytometry. 5 × 10^5^ target cells were incubated at 2–8 °C for 60 min at the indicated catumaxomab concentrations. To analyze the trifunctional binding of catumaxomab different target cells were used: Jurkat cells (ATCC, USA) for binding to CD3, BFTC-905 bladder cancer cells [16] (DSMZ, Germany) for binding to EpCAM, and the monocytic cell line THP-1 (ATCC, USA) that expresses Fcγ RI and IIa. 5 × 10^6^ target cells per ml were resuspended in either PBS buffer or urine samples of healthy donors at final concentrations of 10 and 90% vol. Then, cells were washed two times and cell-bound catumaxomab was detected with F(ab´)2-rat-anti-mouse IgG (H + L)-FITC (Jurkat cells) or mouse-anti-rat IgG (H + L)-FITC (BFTC-905 cells), or F(ab´)2-goat-anti-rat IgG -FITC (THP-1 cells) (Jackson Immuno Research, USA). Stained cells were detected using a FACS-Calibur cytometer (Becton Dickinson, USA).

Catumaxomab-mediated killing of EpCAM-positive BFTC-905 bladder cancer cells was evaluated in an allogeneic cytotoxicity assay [9]. Thereby, the “killing activity” of catumaxomab in PBS buffer control was compared to its activity in samples containing 10% vol urine. Urine samples from each three different male and female donors were tested and freshly prepared PBMC of the same donor were mixed with BFTC 905 tumor cells and the indicated catumaxomab amounts. The effector to target ratio was 5:1. Graph Pad Prism software (Version 5.04) was used for graphical visualization and dose–response curve presentation. Non-linear asymmetric five-parameter curve fitting was applied and EC_50_, top and bottom values and their corresponding 95% confidence intervals were calculated. Goodness of fit as determined by R square was in all cases > 0.98. Outliers are presented in the corresponding figures.

### Detection and quantification of tumor cells in patient urine samples

Midstream urine samples of the patients were analyzed for EpCAM-positive tumor cells before and after the treatment. EpCAM-positive tumor cells were detected by an established and described immunocytochemistry protocol [17]. Briefly, cells in 30 ml urine were centrifuged on cytospins. Slides were double-stained for EpCAM and cytokeratin. EpCAM staining was performed with the EpCAM-specific antibody HO-3 [18] directly labeled with Alexa Fluor 594 Texas Red. For cytokeratin staining the anti-cytokeratin 8, 18, 19 antibody A45B-B3 together with the corresponding Alexa Fluor 488-labeled secondary anti-mouse IgG1 detection antibody (Molecular Probes, USA) were used. All cytospins were analyzed by a computerized image analysis system (MDS, Applied Imaging) counting double stained cells.

### HAMA response, cytokine measurement and systemic catumaxomab detection

HAMA (human anti-mouse antibodies) were quantified by using the medac ELISA kit (medac, Hamburg, Germany) following the instructions of the manufacturer. Concentrations below 40 ng/ml are considered as negative. Cytokine levels in plasma samples were analyzed by using the Luminex system 200 (Luminex, TX, USA) together with the premixed 8-plex fluorokine X-Map kit (R&D Systems, MN, USA) comprising the cytokines IL-2, IL-4, IL-6, IL-8, IL-10, IL-17, IFN-γ and TNF. Samples were collected at the indicated time points, stored at -20 °C and measured in batch. Samples collected on treatment days were taken before antibody instillation and 24 h later. The detection limit of cytokines was 3.2 pg/ml. Systemic catumaxomab concentrations were measured by ELISA as described previously [19]. The quantification limit of the ELISA method is 125 pg/ml.

## Results

### Binding and cytotoxic activity of catumaxomab in urine milieu

**Fig. 1:**
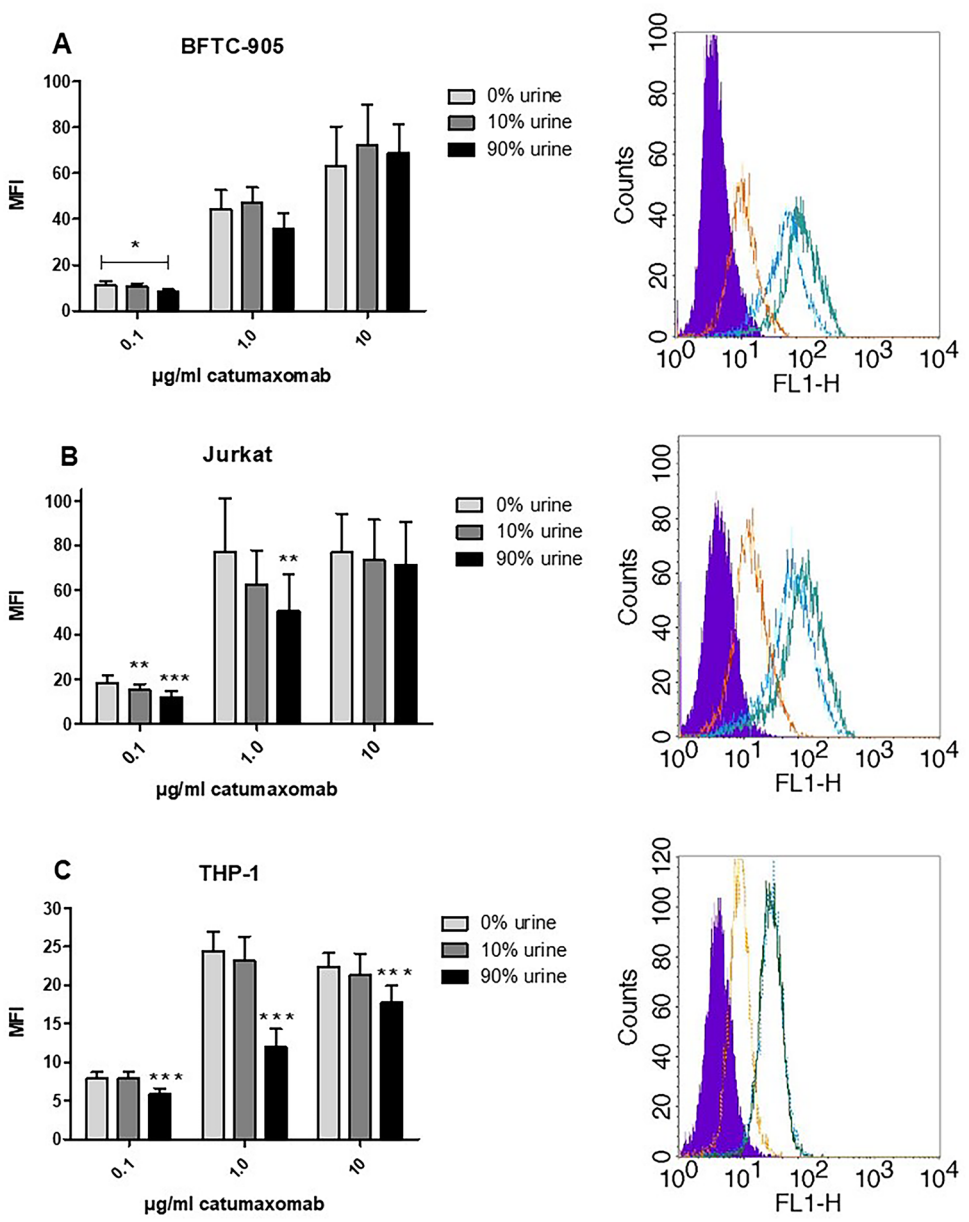
Trifunctional binding activity of catumaxomab in urine samples. Binding of catumaxomab to the bladder carcinoma cell line BFTC-905 (**a**), to CD3-positive Jurkat cells (**b**), or to Fcγ RI/IIa expressing THP-1 cells (**c**) at concentrations of 0.1, 1.0, and 10 µg/ml and in the presence of different urine amounts (0,10, 90% volume) is shown. Each column represents the mean value of six individual measurements (means of double determinations) with urine samples of three healthy male and three healthy female donors. Error bars indicate standard deviation. Asterisks show significant differences between 0% and other urine groups (t-test, * p ≤ 0.05; ** *p*  ≤ 0.01; *** *p*≤ 0.001). MFI = mean fluorescence intensity. On the right hand side, flow cytometry overlays of binding results from one representative donor at 10% urine concentration are included. Different colors indicate cell binding at 10 µg/ml (green line), 1 µg/ml (blue line), or 0.1 µg/ml (orange line) catumaxomab concentration. The filled purple histogram represents the negative control without catumaxomab, but secondary detection antibody only

Catumaxomab was able to effectively bind to all of its target antigens even in the presence of 90% vol urine (Fig. 1a, b, c). A significant decrease in binding was mainly observed at low antibody concentrations of 0.1 µg/ml, whereas at intermediate (1 µg/ml) and at saturating antibody concentrations (10 µg/ml) the diminishing effect was less pronounced or even absent. There was no difference between urine samples from male or from female donors despite different mean pH values (pH 5.3 (male) versus pH 6.7 (female), data not shown).

**Fig. 2:**
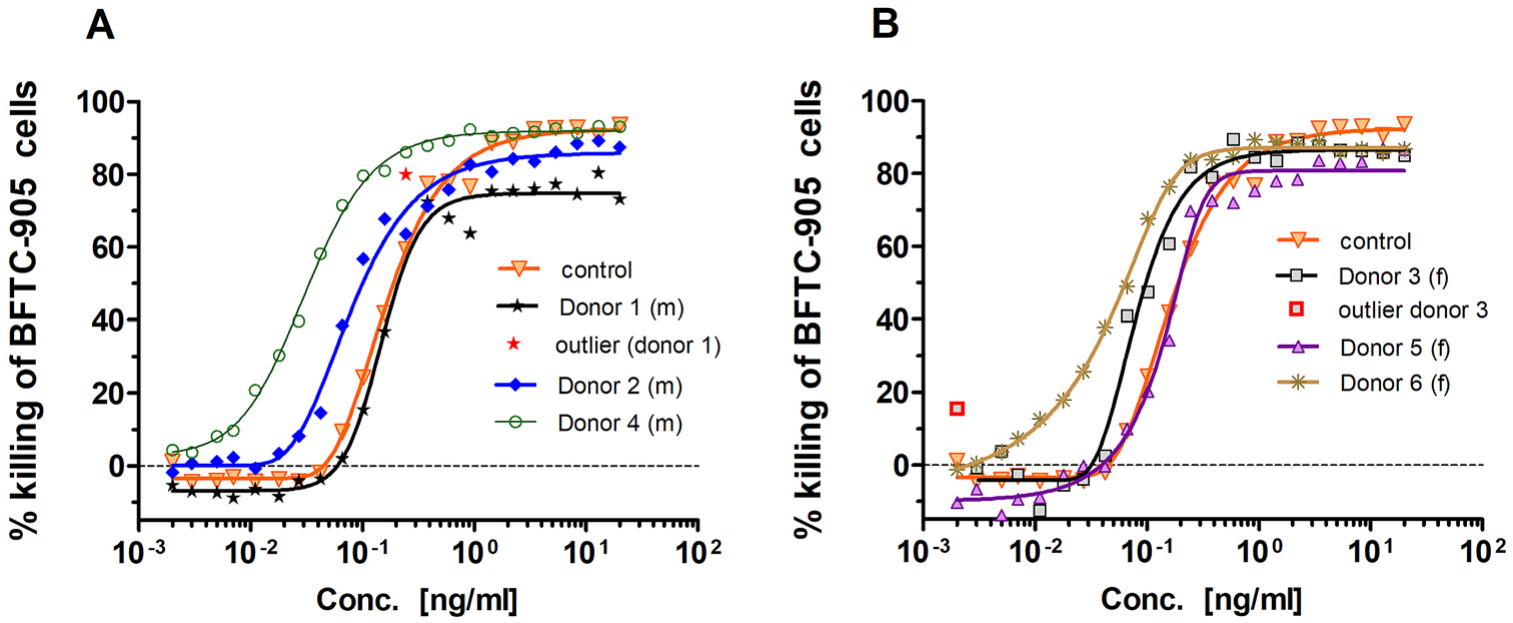
Biological anti-tumor activity of catumaxomab in urine samples. No negative influence was observed on catumaxomab mediated killing of targeted bladder cancer cells BFTC-905 after the addition of 10% vol urine. Each three different urine samples from healthy male (**a**) and female donors (**b**) were tested and compared to buffer control (PBS). 22 different antibody concentrations ranging from 20 ng/ml to 0.002 ng/ml were applied and resulting dose response curves are displayed. Mean results of double determinations are shown; Calculated EC_50_ values ranged from 0.03 to 0.15 ng/ml (buffer control 0.16 ng/ml)

Catumaxomab-induced cytotoxic activity against bladder cancer cells was tested in a urine milieu of 10% vol. (three urine samples of different male and female healthy donors). Catumaxomab maintained its killing activity in the presence of urine samples and no sex-related difference was observed (Fig. 2). The cytotoxic activity of catumaxomab was comparable to buffer control (EC_50_ = 0.16 ng/ml) or even more effective in urine samples with EC_50_ values ranging between 0.03 ng/ml and 0.15 ng/ml.

### Treatment results of patient 1

The first patient was a 70 years old woman with urothelial cell carcinoma (pTa, G2) initially diagnosed in 2004. Between 2005 and 2015, she was suffering from five local recurrences (pTa, G1, singular and monolocular) which could be removed by TUR-B. After a further tumor recurrence in 2015, the patient denied the TUR-B for personal reasons. Having confirmed EpCAM-positive tumor cells she was treated with six doses of catumaxomab starting with 20 µg up to 100 µg (total amount of antibody 470 µg).

The antibody was well tolerated without adverse events such as fever or flu-like symptoms which correlated with low systemic cytokine levels, which are in contrast elevated when catumaxomab was administered intraperitoneally [20] or intravenously [21]: No IL-2, IL-4, IL-10, IL-17 or IFN-γ was traceable in plasma (Table 1). Only very low and transient amounts of IL-6 (10-13 pg/ml) were measured after the first and second instillation. Plasma concentrations of catumaxomab remained below the quantification limit of 125 pg/ml. Induction of human anti-mouse antibodies (HAMA) did not occur up to 14 days after the end of the antibody therapy (Table 1).

**Table 1:** Immunomonitoring results of patient 1

Day	Catumaxomab (µg)	IL-2, 4, 10, 17 IFN-γ^§^(pg/ml)	IL-6 (pg/ml)^§^	IL-8 (pg/ml)^§^	TNF (pg/ml)^§^	Systemic Catu (pg/ml)*	HAMA^+^	Number of tumor cells^#^
0	20	< 3.2	< 3.2	8	7	< 125	Neg	23
1		< 3.2	< 3.2	9	6	< 125		
7	50							
8		< 3.2	13	< 3.2	< 3.2	< 125		
14	100	< 3.2	10	< 3.2	< 3.2	< 125	Neg	
21	100							
22		< 3.2	< 3.2	< 3.2	< 3.2	< 125		
28	100	< 3.2	< 3.2	< 3.2	< 3.2	< 125	Neg	
29		< 3.2	< 3.2	< 3.2	< 3.2	< 125		
35	100	< 3.2	< 3.2	< 3.2	< 3.2	< 125	Neg	12
36		< 3.2	< 3.2	< 3.2	< 3.2	< 125		
49		< 3.2	< 3.2	< 3.2	< 3.2	< 125	Neg	0
148								0
211								0
339								0
456								0
631								1
701								0

*Lower limit of quantification for plasma samples = 125 pg/ml

^§^ Detection limit of cytokines in plasma samples = 3.2 pg/ml

^+^ Values below 40 ng/ml are considered negative;

^#^ 30 ml urine samples were analyzed

**Fig. 3:**
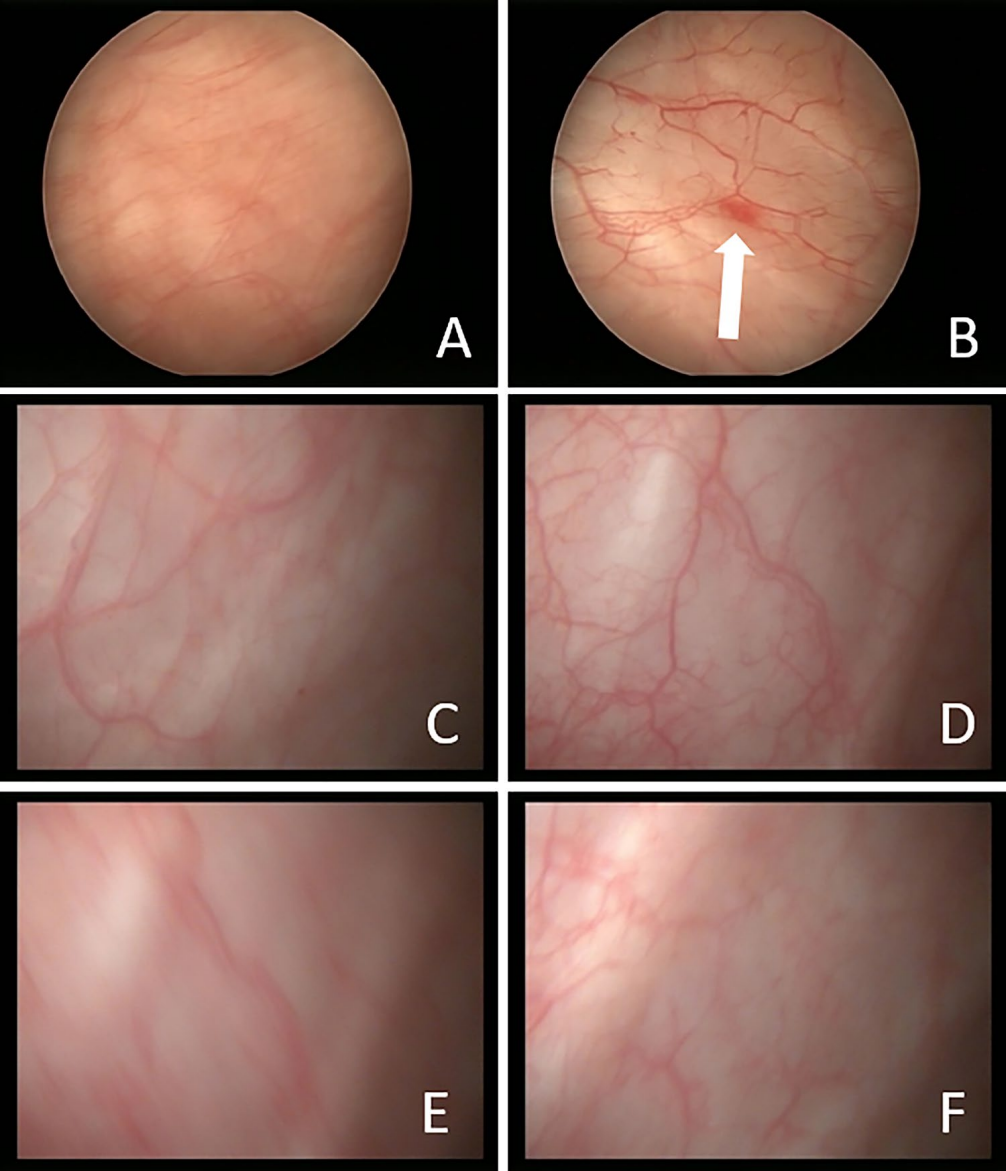
Endoscopic evaluation before and after catumaxomab treatment. Endoscopic images of the bladder of patient 1 at different magnifications two weeks before (**a**, **b**) and two weeks after (**c**–**f**) treatment with catumaxomab are shown. Arrow in B points to a growing papillary structure that disappeared after one treatment cycle with catumaxomab (**c**–**f**)

The number of EpCAM-positive tumor cells dropped from 23 to 0, 14 days after the last instillation and follow-up samples were still negative for tumor cells until day 701 with the exception of day 631 where one tumor cell was found (Table 1). Endoscopic imaging of the bladder confirmed this finding. A growing papillary structure, typical for superficial bladder cancer, was imaged two weeks before the start of catumaxomab treatment and was not detectable two weeks after the last catumaxomab application. Apart from a slight inflammation the mucosa appeared completely normal (Fig. 3). The patient remained relapse-free confirmed by cystoscopy and urine cytology every three months. Last follow-up visit was at 32 months. Further regular control visits were not possible due to occurring dementia. A last contact, 45 months after treatment showed inconspicuous urine cytology with now signs of microhematuria.

### Treatment results of patient 2

A 72 years old man with urothelial cell carcinoma (pTa, G2) that was first diagnosed in June 2004 and in October 2014 a papillary growing neoplasia with positive FISH-test and dysplastic urothelial cells was detected during an endoscopic control visit. Because of several cardiovascular and other comorbidities (hepatopathy) a proposed TUR-B was refused by the patient. Alternatively, a first treatment cycle with six weekly, intravesically administered doses of catumaxomab antibody was started in March 2015. Urine cytology displayed various urothelial cells with enlarged, hyperchromatic cell nuclei and a nucleus/plasma ratio changed in favor of the cell nuclei. Ten days after treatment no atypical cells were observed. Endoscopic follow-up controls and urine cytology remained negative for approximately six months. An unclear inflammatory change of the bladder mucosa with a positive Urovysion test (Abbott) implicated a relapse afterwards. The patient received a second treatment cycle consisting of seven weekly instillations of 1 × 50 and 6 × 100 µg of catumaxomab. EpCAM-positive tumor cells were detectable in the urine but continuously dropped from 111 to 0, 52 days after the last instillation of the second treatment cycle (Table 2). Eleven tumor cells were found at day 770 but endoscopic controls every three months remained inconspicuous for a follow-up period of 25 months. After 36 months the patient progressed with a diagnosed, histopathologic confirmed urothelial cell carcinoma in the right renal pelvis. In view of several comorbidities an operative treatment was not performed and the patient died shortly after diagnosis due to cardiovascular complications.

**Table 2:** Immunomonitoring results of patient 2

Day	Catumaxomab (µg)	IL-2, 4, 10, 17 IFN-γ^§^(pg/ml)	IL-6 (pg/ml) §	IL-8 (pg/ml)^§^	TNF (pg/ml)^§^	Systemic Catu (pg/ml)*	HAMA ^+^	Number of tumor cells ^#^
*First treatment cycle*								
0	20	< 3.2	< 3.2	9	9	< 125	40	Not evaluable**
7	50	< 3.2	< 3.2	8	7	< 125	67	
8		< 3.2	< 3.2	10	7	214		
14	100					162		
15		< 3.2	16	9	< 3.2			
21	100	< 3.2	13	9	< 3.2	< 125	198	
22		< 3.2	13	10	< 3.2	< 125		
28	100	< 3.2	< 3.2	< 3.2	< 3.2	< 125	163	
29		< 3.2	< 3.2	< 3.2	< 3.2	170		
35	100	< 3.2	< 3.2	< 3.2	< 3.2	< 125	60	Not evaluable**
36		< 3.2	< 3.2	< 3.2	< 3.2	< 125		Not evaluable**
*Second treatment cycle*								
225	50	< 3.2	< 3.2	< 3.2	< 3.2	< 125	159	
226		< 3.2	< 3.2	< 3.2	< 3.2	< 125		111
232	100	< 3.2	< 3.2	< 3.2	< 3.2	< 125	63	
233		< 3.2	< 3.2	< 3.2	< 3.2	< 125		83
239	100	< 3.2	< 3.2	< 3.2	< 3.2	< 125	63	
240		< 3.2	< 3.2	< 3.2	< 3.2	< 125		26
246	100	< 3.2	< 3.2	< 3.2	< 3.2	< 125	64	
247		< 3.2	< 3.2	< 3.2	< 3.2	< 125		27
253	100	< 3.2	< 3.2	< 3.2	< 3.2	< 125	154	
254		< 3.2	< 3.2	< 3.2	< 3.2	< 125		8
260	100	< 3.2	< 3.2	< 3.2	< 3.2	< 125	281	
261		< 3.2	< 3.2	< 3.2	< 3.2	< 125		6
267	100	< 3.2	< 3.2	< 3.2	< 3.2	< 125		
268		< 3.2	< 3.2	< 3.2	< 3.2	< 125		4
319								0
464								0
770								11

*Lower limit of quantification for plasma samples = 125 pg/ml

^+ ^ Values below 40 ng/ml are considered negative

^#^ 30 ml urine samples were analyzed;

** samples not evaluable due to high cell debris background

^§^Detection limit of cytokines in plasma samples = 3.2 pg/ml

Catumaxomab treatment was well tolerated without signs of fever, inflammation or cytokine-induced side effects. Apart from an intermediate detection of IL-6 (16-13 pg/ml) no cytokines were detected (Table 2). Catumaxomab slightly above the quantification limit of 125 pg/ml was traceable in plasma after the second and the fifth application and low HAMA levels were measured during the first and second treatment cycles (Table 2).

## Discussion

In spite of new treatment options like PD-1/PD-L1 checkpoint inhibitors [22–24], advanced and metastasized bladder cancer remains a fatal disease. Fortunately, a majority of about 75% of diagnosed bladder cancers is local and non-muscle-invasive [25]. Following TUR-B, recommended prophylactic and maintenance BCG therapy [26–28] is associated with severe and painful inflammation-caused side effects and about 8–20% of patients suspend the treatment [4, 5] and 30–40% of patients do not respond to the therapy [29]. For BCG-refractory, -unresponsive or -intolerant patients radical cystectomy often represents the ultima ratio. impacting the quality of life.

Due to its expression pattern in TCC, EpCAM is a clinically relevant antigen target for immunotherapy of bladder cancer [6, 7]. In an in vitro urinary environment catumaxomab showed a robust binding to all three target antigens or receptors, respectively (EpCAM, CD3 and Fcγ RI/IIa; Fig. 1). Inhibitory effects of urine were mainly seen at lower antibody concentrations (< 1 µg/ml) and were compensated by the application of higher local antibody amounts. In accordance with the binding results, catumaxomab-induced cytotoxicity against EpCAM-positive bladder cancer cells was not diminished in 10% vol. urine samples (Fig. 2). Higher urine concentrations might also be tolerable but could not be tested because of the disturbing influence of the urine itself on the growth of the tumor cells. These non-clinical results suggest that catumaxomab might indeed be immunologically active in the bladder of cancer patients assuming that sufficient immune effector cells are present. Potentially recruited effector cells may derive from the urinary fluid or from immune cells infiltrating the tumor lesions. By virtue of its trifunctional mode of action catumaxomab strongly activates not only T cells but also accessory immune cells which may lead to the recruitment of additional effector cells as shown previously for malignant ascites [17].

This hypothesis was tested in two individuals with a history of recurrent NMIBC who denied further TUR-B for personal and medicinal reasons.

After intravesical administration of catumaxomab no EpCAM-positive tumor cells were found any more in the urine after one, respectively two treatment cycles (Tables 1 and 2). In one patient, the dissolving of a neoplastic, papillary structure could be confirmed by endoscopic imaging (Fig. 3). Follow-up endoscopic re-evaluations every three months confirmed an ongoing recurrence-free interval of 32 and 25 months. These data indicate that catumaxomab might be clinically effective and safe in the treatment of recurrent NMIBC.

Total amounts of 470 µg and 1120 µg catumaxomab, respectively, could be applied without any signs of toxicity in contrast to i.p. administered catumaxomab. where the maximum tolerated dose (MTD) was found at 10-20-50 and 200 µg [30]. The predominant catumaxomab-induced side effects like fever, chills, headache and vomiting are mediated by the release of cytokines [30]. No relevant cytokine levels could be detected in the plasma of the patients (Tables 1 and 2) indicating no systemic distribution of catumaxomab after instillation into the bladder. Accordingly, the lack of HAMA development may allow the repeated application of the drug without loss of efficacy. This was demonstrated for patient 2 who received a second treatment cycle and thereafter showed a complete elimination of EpCAM-positive tumor cells in the urine.

In conclusion, the intravesical application of catumaxomab in two patients with recurrent NMIBC was feasible and safe and demonstrated first signs of efficacy. Therefore, a phase I clinical study has been started to investigate safety and tolerability of intravesically applied catumaxomab in patients with high risk NMIBC (Eudract: 2019-002,850-22). For further clinical development also combination treatments of catumaxomab and BCG or immune checkpoint inhibitors have to be considered.

## Data Availability

The datasets used and analyzed during the current study are available from the corresponding author on reasonable request.

## Abbreviations

**BCG**: Bacillus Calmette–Guérin
**EpCAM**: Epithelial cell adhesion molecule
**HAMA**: Human anti-mouse-antibody
**i.p**.: Intraperitoneal
**NMIBC**: Non-muscle-invasive bladder cancer
**PBMC**: Peripheral blood mononuclear cells
**TCC**: Transitional cell carcinoma
**TUR-B**: Transurethral resection of the tumor
